# Baduanjin Mind-Body Intervention Improves the Executive Control Function

**DOI:** 10.3389/fpsyg.2016.02015

**Published:** 2017-01-13

**Authors:** Tingting Chen, Guang H. Yue, Yingxue Tian, Changhao Jiang

**Affiliations:** ^1^Department of Psychology, Capital Normal UniversityBeijing, China; ^2^Human Performance and Engineering Research, Kessler Foundation, West OrangeNJ, USA; ^3^Department of Psychology, Rice University, HoustonTX, USA; ^4^Beijing Key Lab of Physical Fitness Evaluation and Tech Analysis, Key Laboratory of Sports Ability Evaluation and Research Comprehensive Laboratory of General Administration of Sport, Capital University of Physical Education and SportsBeijing, China

**Keywords:** Baduanjin exercise, near-infrared spectroscopy, flanker task, mood state, executive function, left prefrontal cortex

## Abstract

This study aims at comparing the effects of the Baduanjin mind-body (BMB) intervention with a conventional relaxation training program on enhancing the executive function. The study also attempts to explore the neural substrates underlying the cognitive effect of BMB intervention using near-infrared spectroscopy (NIRS) technique. Forty-two healthy college students were randomly allocated into either the Baduanjin intervention group or relaxation training (control) group. Training lasted for 8 weeks (90 min/day, 5 days/week). Each participant was administered the shortened Profile of Mood States to evaluate their mood status and the flanker task to evaluate executive function before and after training. While performing the flanker task, the NIRS data were collected from each participant. After training, individuals who have participated in BMB exercise showed a significant reduction in depressive mood compared with the same measure before the intervention. However, participants in the control group showed no such reduction. The before vs. after measurement difference in the flanker task incongruent trails was significant only for the Baduanjin intervention group. Interestingly, an increase in oxygenated hemoglobin in the left prefrontal cortex was observed during the Incongruent Trails test only after the BMB exercise intervention. These findings implicate that Baduanjin is an effective and easy-to-administering mind-body exercise for improving executive function and perhaps brain self-regulation in a young and healthy population.

## Introduction

As an important concept of traditional Chinese medicine theories, mind-body training emphasizes the interaction between the brain, the mind, and the body (Chan et al., 2009a), with *qigong, tai chi*, and *yoga* being the most frequently used techniques. The fundamental assumption is that individuals can regulate breathing, heart, and body activities by their own thoughts, resulting in enhancement in physical and mental health (Cheng, 2015). A growing number of empirical studies have reported that doing mind-body exercise regularly has a positive impact on emotional and psychological processes in clinical and normal populations (Sandlund and Norlander, 2000; Chou et al., 2004; Wang et al., 2004). To take a simple example, Yoga practice was reported to lead to improvements in quality of life, psychological functioning, and symptom indices in female cancer survivors. Interestingly, Yoga practice was associated with a linear increase in associative attention and positive affective valence (Mackenzie et al., 2014). Another randomized controlled study showed therapeutic benefits on reducing intake of antidepressants, improving depressive symptoms, and enhancing attentional abilities in patients with depression after a 10-session Chinese Chan-based Dejian mind-body intervention (DMBI) (Chan et al., 2013). Similar effects were also observed on primary school children after 4 months of DMBI (Brown et al., 1995), healthy adults after 1-month Shaolin Dan Tian Breathing (DTB) (Chan et al., 2011a), college students (Liu et al., 2008; Chen and Liu, 2013), and elderly individuals (Chen, 2013; Zhang and Ai, 2013) after 12 weeks of Eight-Brocade Exercise.

Apart from encouraging effects of mind-body training on emotional problems and psychological well-being, some empirical data have also suggested that mind-body training has a positive impact on cognitive function in clinical samples and healthy aging. In clinical practice, the DMBI helped the chronic epileptic patient enhance language, memory, attention, behavioral initiation, emotional control, and social functioning; and assisted low functioning patients with autism improve inhibitory control, cognitive flexibility, and memory functioning (Chan et al., 2009b, 2011b). A recent study (Chattha et al., 2008) reported that an 8-week integrated approach yoga therapy (IAYT) effectively elevated attention, concentration, mental balance, verbal retention, and recognition abilities in climacteric women compared with those participated in a conventional physical exercise program. For older adults, although age-related changes in cognitive function, such as declines in executive function, information processing speed, and attention are common, the benefits of mind-body exercise on these abilities in older adults are also well-documented. For instance, as a form of mind-body exercise, Tai Chi appears to help maintain executive function, language, learning and memory, and subjective memory in older adults (Miller and Taylor-Piliae, 2014).

Cognitive function refers to a person’s ability to process thoughts, memory, learning new information, speech, and reading comprehension (Royall et al., 2002). Together they are key components of both “cognitive control” and “executive function.” Executive function (EF) refers to the higher-order cognitive control process for the attainment of a specific goal (Moriguchi and Hiraki, 2013), and broadly encompasses a set of cognitive skills that are responsible for the planning, initiation, sequencing, and monitoring complex goal directed behavior (Royall et al., 2002). Components of executive function are measured by a variety of tests of abstraction and mental control, such as Stroop Test, Trail Making Test (TMT), Oral Reading Span Test (RST), and so on. Extensive neuroimaging studies have reported that better performance on these tests of executive functions was associated with larger prefrontal cortical volume and cortical thickness (Garavan et al., 2002; Moriguchi and Hiraki, 2013; Yasumura et al., 2014; Yuan and Raza, 2014). The unique structure and connectivity pattern of prefrontal cortex functionalized itself the only cortical region capable of integrating motivational, mnemonic, emotional, somatosensory, and external sensory information into unified, goal-directed action (Royall et al., 2002). Individual’s cognitive and neural development may be sensitive to physical activity (Yanagisawa et al., 2010; Davis et al., 2011; Verburgh et al., 2014), but few studies have investigated the functional benefits and underlying training-induced neural plasticity contributing to the benefits.

The purpose of this study was to examine the effect of an 8-week Baduanjin mind-body (BMB), a traditional Chinese mind-body exercise, intervention on changing executive function and NIRS-measured prefrontal cortex activity in college students. NIRS is an emergent imaging technique for investigating cortical hemodynamic response. Since oxygenated hemoglobin (oxy-Hb) and deoxygenated hemoglobin (deoxy-Hb) have different absorption spectra in the infrared range, changes in oxy-Hb and deoxy-Hb can be calculated by detecting infrared light at different wavelengths on the skull. In general, enhanced oxy-Hb and reduced deoxy-Hb are associated with regional cortical activation (Yasumura et al., 2014). NIRS is noninvasive and robust against body movement, and has been validated as a suitable technique for investigating neural mechanisms in psychological experiments (Tsujii et al., 2012). Baduanjin, a form of *qigong*, can slow age-related memory decline (Wang, 2007) and has a positive effect on lowering blood pressure, blood lipid, and inflammatory factors, which are risk factors for cognitive impairment (Mei et al., 2012; Xiong et al., 2015). Zheng et al. (2016) have recently shown that the Baduanjin exercise is beneficial in maintaining or even improving both global cognitive function and specific domains of cognition including memory, processing speed, executive function, attention and verbal learning and memory in older adults with mild cognitive impairment. Consequently, we hypothesized that short-term Baduanjin training could be beneficial to executive function in normal people, and also be reflected in neural activity level.

## Materials and Methods

### Participants

**FIGURE 1:**
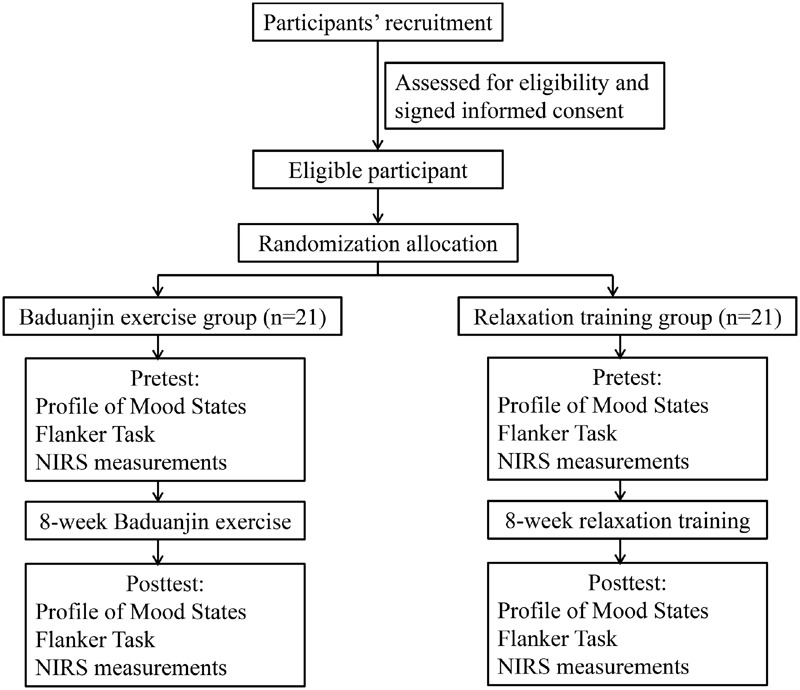
**Flow diagram of study design**.

Forty-two right-handed undergraduate or graduate students (26 females; mean age 22.5 ± 2.0 years, range 19–26 years; body mass 52.2 ± 6.8 kg, height 165.3 ± 4.6 cm) took part in this experiment and were paid for their participation. Individuals were asked to complete a short questionnaire on their emotional and physical conditions. Individuals with any one of the following conditions were excluded from the study: (i) history of psychiatric, neurological, musculoskeletal disorders, or substance abuse; (ii) history of BMB or any other mind body training. The participants were randomly assigned into the BMB intervention group or relaxation exercise (control) group (see **Figure 1**). Paired *t*-test showed that no statistical differences were observed in any of the biographical variables indicated above between the two groups (*P*s > 0.05). All subjects had normal or corrected-to-normal vision, and normal color vision. The Institutional Review Board at Capital University of Physical Education and Sports, where the experiments were performed, approved the study. All participants provided a written informed consent prior to their participation.

### Training

The intervention group received 8 weeks of BMB exercise training under the guidance of an experienced coach, with a frequency of 5 days a week and 90 min a day including 10 min warm up, 70 min Baduanjin training and 10 min cool down. The BMB exercise consists of eight movements for limbs, body-trunk, and eye movements. These eight movements are“holding the hands high with palms up to regulate the internal organs,” “posing as an archer shooting both left-and right-handed,” “holding one arm aloft to regulate the functions of the spleen and stomach,” “looking backwards to prevent sickness and strain,” “swinging the head lowering the body to relieve stress,” “moving the hands down the back and legs and touching the feet to strengthen the kidneys,” “thrusting the fists and making the eyes glare to enhance strength,” and “raising and lowering the heels to cure diseases”, respectively (Cheng, 2015). This movement set aims not only at strengthening musculoskeletal fitness (Koh, 1982) and circulation together with a qi breathing training, but also regulating emotions, representing body-mind effectiveness. On the other hand, the control group received 8 weeks of progressive muscle relaxation training with the same frequency and session duration, which helps the participants achieve physical and mental relaxation and calmness (Tang et al., 2007, 2010). Participants were taught in a standard procedure: beginning with some deep breaths with closed eyes, followed by relaxation exercises of different muscle groups over the face, head, shoulders, arms, legs, chest, back, and abdomen, guided by an athletic trainer and compact disk. Participants were to inhale when tensing the muscles, exhale when relaxing, and concentrate on the sensation of relaxation, such as the feelings of warmth and happiness.

### Measures

Each participant in the intervention and the control group was administered the baseline assessment within 2 weeks before the training, and the post-assessment within 3 days after the training. Participants were also administered the shortened Profile of Mood States (POMS) to evaluate their mood status. Executive function was measured using the flanker task. During completing the flanker task, the NIRS data were collected from each participant.

#### Profile of Mood States

The POMS (short version) is a checklist consisting of 40 adjectives that are rated on a scale from 0 (not at all) to 4 (extremely) according to how subjects feel. The items produce scores for seven subscales (score ranges in parentheses): Tension-Anxiety (six items), Anger-Hostility (seven items), Fatigue-Inertia (five items), Depression-Dejection (six items), Vigor-Activity (six items), Confusion-Bewilderment (five items), and Self-esteem (five items). Standard procedures were used to score the subscales of the original POMS (McNair et al., 1981). Total Mood Disturbance (TMD) scores were then computed using the formula Depression-Dejection + Tension-Anxiety + Anger-Hostility + Fatigue-Inertia + Confusion-Bewilderment - (Vigor-Activity + Self-esteem) + 100 (Andrykowski et al., 1990; Andrykowski and Hunt, 1993). High TMD scores indicate negative affective states for all scales except vigor and self-esteem, which is a positive mood measure (Curran et al., 1995).

#### Flanker Task

Participants completed congruent and incongruent conditions of the flanker task (Eriksen and Eriksen, 1974). Congruent trials were those in which the target arrow was flanked by the same arrow (e.g., > > > > > or < < < < <). Incongruent trials were those in which the target arrow was flanked by the opposing response arrow (e.g., > > < > > or < < > < <) (White et al., 2011). An array of arrows were presented on a computer monitor from a distance of 1 m with visual angles of 1.7° and 3.7° in the vertical and horizontal directions, respectively. The stimuli were 7.62-cm-tall white arrows presented focally on a black background in a random order for 200 ms with an inter-stimulus interval of 1,000 ms from stimulus offset to onset.

Participants were asked to press “f” button when the central arrow in a display faced left, and “j” button when the central arrow faced right. The task was presented in a randomized block design consisting of four blocks congruent trials, four blocks of incongruent trials with 30 trials per block (a total of 240 trials). Each block lasted 30 s with a 30-s resting period separating the adjacent congruent and incongruent blocks.

#### NIRS Measurements

Each subject sat on a comfortable chair in a lighted room with eyes open throughout each measurement. NIRS measurements was completed immediately before and after the Eight Brocades. Changes in blood flow were measured by using a 44-channel NIRS system (ETG-4000: Hitachi Medical Corporation, Tokyo, Japan). Two equal probe sets with 3 × 5 arrays of light emitters and detectors were symmetrically placed on scalp over the prefrontal cortex. A measuring point of activation was defined as the region between one emitter and one detector. One array consisted of 22 channels and covered an area of 12 cm × 6 cm. This apparatus can measure the relative concentrations of oxy-Hb and deoxy-Hb at 44 measurement points in two areas of 15 cm × 6 cm each. The location of each shell was determined on the international 10–20 system. The most inferior medial channels of the left and right prefrontal lobes were located at Fp1and Fp2, respectively. Concentration changes in oxy-Hb and deoxy-Hb were calculated by using the difference in absorbance based on a modified Beer–Lambert law (Shibuya-Tayoshi et al., 2007).

## Results

### Mood-Enhancing Effect

A 2 × 2 repeated measures ANOVA was performed on TMD score before and after treatment (time) between the two groups. A significant main effect of Time was found, [*F*(1,40) = 14.23, *p* < 0.01]. The interaction between time (before or after treatment) and group (intervention or control group) was also significant [*F*(1,40) = 20.24, *p* < 0.001]. The two groups were comparable on the level of depressive mood at baseline as measured by their TMD score [*t*(40) = 1.78, *p* = 0.08]. For each group, statistical analysis was performed using paired *t*-test to compare the post-training mood states with the pre-training mood states within group. Within-group comparisons revealed that TMD score decreased significantly after BMB exercise training [*t*(20) = 6.45, *p* < 0.001] but the change was not significant in the control group [*t*(20) = -0.47, *p* = 0.64] (see **Table 1**).

**Table 1:** Total Mood Disturbance (TMD) score with standard errors before and after treatment between the two groups (intervention vs. control).

	Intervention group	Control group
Before training	117.95 ± 3.22	110.86 ± 2.32
After training	98.43 ± 2.48	112.57 ± 3.32

### Flanker Task Performance

**FIGURE 2:**
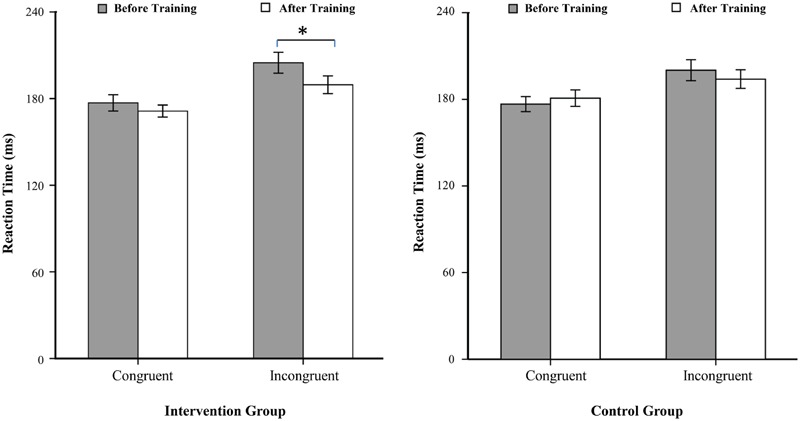
**Reaction times, with standard errors, as a function of the experimental conditions.** ^∗^*p* < 0.05.

A 2 (time: before vs. after) × 2 (flanker congruency: congruent vs. incongruent) × 2 (group: intervention vs. control) analysis of variance (ANOVA) revealed the following significant main effects: time [*F*(1,40) = 7.39, *p* < 0.01] and flanker congruency [*F*(1,40) = 108.31, *p* < 0.001]. Importantly, the time × group and the time × flanker congruency interactions were also significant, *F*(1,40) = 5.03, *p* < 0.05, and *F*(1,40) = 11.77, *p* < 0.001. No other significant main effects or interactions were observed (**Figure 2**).

Separate ANOVAs, with the time (before vs. after) and the flanker congruency (congruent vs. incongruent) as two within-participant factors, were conducted for trials in the intervention group and for trials in the control group. For the control group, only the main effect of flanker congruency was significant [*F*(1,20) = 40.94, *p* < 0.001]. The main effect of time was not significant [*F*(1,20) = 0.31, *p* = 0.59], nor was the interaction between time and flanker congruency [*F*(1,20) = 4.20, *p* = 0.055]. Planned pairwise comparisons showed that no significant differences for reaction times were observed between the before and after measurement in the congruent trails [*t*(20) = –1.51, *p* = 0.15], as well as in the incongruent trails [*t*(20) = 1.81, *p* = 0.085]. For the intervention group, however, both the main effect of the time and the main effect of the flanker congruency were significant, *F*(1,20) = 7.55, *p* < 0.05, and *F*(1,20) = 69.97, *p* < 0.001, respectively. Importantly, the interaction between the two factors was also significant [*F*(1,20) = 11.05, *p* < 0.005]. Planned pairwise comparisons showed that reaction times did not differ in the congruent trails between the before and after training [*t*(20) = 1.58, *p* = 0.13], but they did differ in the incongruent trails between before and after training [*t*(20) = 3.39, *p* < 0.005]. Before BMB exercise training, no differences were found for executive networks in two groups (*P*s > 0.05).

### NIRS Response

**FIGURE 3:**
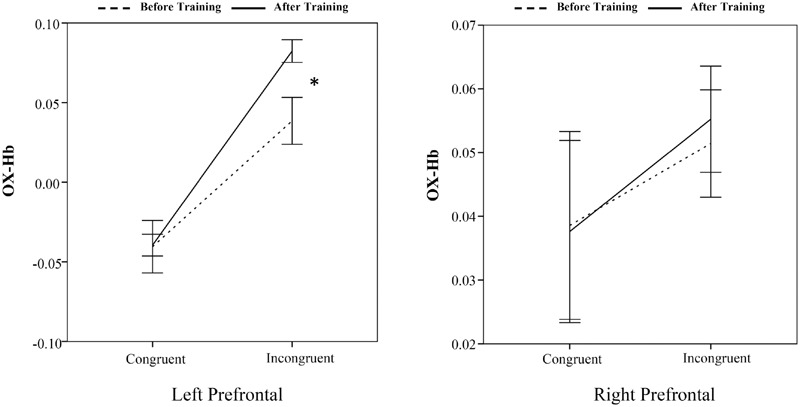
**Baduanjin mind-body (BMB)-related oxyhemoglobin concentration changes during the intervention group performing the flanker task.** After BMB exercise, only the oxyhemoglobin concentration change in left prefrontal lobe for incongruent trails was significant (^∗^*p* < 0.05).

**FIGURE 4:**
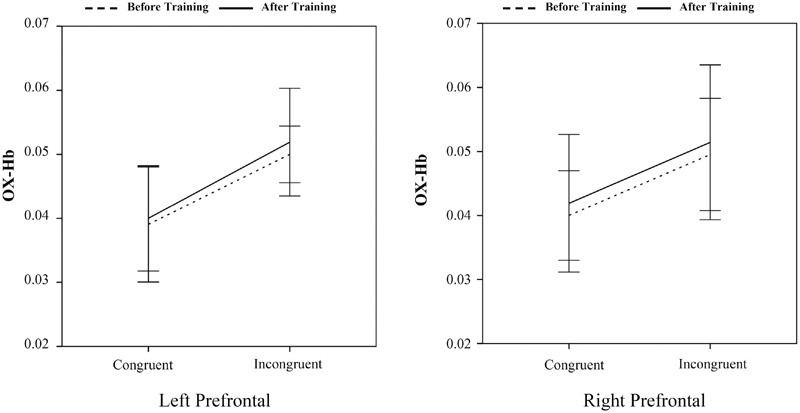
**Oxyhemoglobin concentration changes during the control group performing the flanker task.** No significant (before vs. after)-training differences were found (*P*s > 0.05).

A 2 (time: before vs. after) × 2 (flanker congruency: congruent vs. incongruent) × 2 (group: intervention vs. control) × 2 (frontal lobe: left vs. right) analysis of variance (ANOVA) showed a significant main effect of time [before vs. after, *F*(1,40) = 16.30, *p* < 0.001]. The main effect of frontal lobe (left vs. right) was also significant [*F*(1,40) = 80.13, *p* < 0.001]. More importantly, the time × group, frontal lobe × group, and time × frontal lobe × group interactions were significant, [*F*(1,40) = 15.44, *p* < 0.001, *F*(1,40) = 38.53, *p* < 0.001, and *F*(1,40) = 9.70, *p* < 0.005]. No other main effects or interactions reached significance. Pairwise comparisons, with Bonferroni correction, were conducted. The increased oxy-Hb at the left prefrontal cortex was found only for the incongruent trails after the 8 weeks of BMB exercise [*t*(20) = -2.42, *p* < 0.05]. No other cross (before vs. after)-training differences for the incongruent trails were found (*Ps* > 0.05) (**Figure 3** for the intervention group and **Figure 4** for the control group) (see **Table 2**).

**Table 2:** Statistical results of oxyhemoglobin concentration changes in prefrontal regions before and after treatment between the two groups (intervention vs. control), with standard errors.

	Intervention group				Control group			
	Left prefrontal cortex		Right prefrontal cortex		Left prefrontal cortex		Right prefrontal cortex	
	Congruent	Incongruent	Congruent	Incongruent	Congruent	Incongruent	Congruent	Incongruent
Before training	-0.041 ± 0.016	0.038 ± 0.014	0.038 ± 0.014	0.051 ± 0.008	0.039 ± 0.008	0.050 ± 0.004	0.040 ± 0.007	0.049 ± 0.008
After training	-0.039 ± 0.007	0.082 ± 0.007	0.037 ± 0.014	0.055 ± 0.008	0.040 ± 0.008	0.052 ± 0.008	0.041 ± 0.011	0.051 ± 0.012

## Discussion

This study revealed that BMD exercise may have a significant mood-enhancing effect on college students even with just 8 weeks of training. Specifically, individuals who participated in the BMB exercise showed significant reduction in depressive mood compared with those who participated in the relaxation exercise training (control group). With a short-term intervention, the BMB exercise seemed to be more effective than a relaxation exercise program in improving executive control of college students. This was manifested by the observation that the before vs. after measurement difference in the incongruent trails was significant only for the group trained by the BMB exercise. In addition, the NIRs measures provided insights into the possible neural mechanism that may be associated with the improvement of executive functions. That is, the increased oxy-Hb in the left prefrontal cortex was found for the incongruent trails after 8 weeks of BMB exercise training.

There have been scientific and clinical studies which demonstrated positive effects of mind-body exercise on mood states in clinical and healthy populations (Liu et al., 2008; Chan et al., 2011a; Chen, 2013; Chen and Liu, 2013; Zhang and Ai, 2013). Consistent with these previous studies, no significant difference in mood was detected before training between the two groups. After training, however, the BMB exercise group showed significantly lower TMD scores in negative affect in comparison with the control group. These results suggest that a relatively short-term BMB exercise training can induce higher positive mood and lower negative mood states than a relaxation training. An important component of traditional Chinese *qigong*, Baduanjin exercise can help practitioners to reach coordination between mind and body. When one can internally change his/her own thoughts and behaviors in a coordinated manner, enhancements in emotional and physical health are achieved (Cheng, 2015).

Executive function is a complex concept that includes working memory, overlaps with attention, and requires sensory selection, response selection, and vigilance (Miller and Taylor-Piliae, 2014). Previous studies that examined effects of mind-body interventions on cognitive function in community-dwelling older adults measured components of executive function using a variety of tests that included the Clock Drawing Test (CDT), Color Trails Test (CTT), Digit Span Tests (DS), Digit Symbol Tests (DSym), Color–Word Matching Stroop task (ST), and TMTs (Miller and Taylor-Piliae, 2014). These studies paid attention both to improving clinic treatments, and preventing cognitive impairment (World Health Organization and Calouste Gulbenkian Foundation, 2014) by means of creative measures, but not by focusing on how to enhance the executive function in healthy adults. In addition, in clinical populations, executive functions were often measured using the Functional Independence Measure (FIM), Functional Status Rating System (FSRS) (Chan et al., 2009b), and Behavior Rating Inventory of Executive Function (BRIEF) (Chan et al., 2011b). Self-reports of behaviors and attitudes are strongly influenced by features of the research instrument, the data of which may weaken the results. Therefore, the present study used the flanker task as the standardized tool to explore the effects of BMB exercise on executive function in college students. Findings from the present study support the notion that the BMB exercise is an effective practice for improving executive function in the young and healthy population, indicated by a significant improvement in behavioral responses to the incongruent trials only in the BMB intervention group but not in the control group.

Extensive neuroimaging studies have reported that better performance on the tests of executive functions was associated with larger prefrontal cortical volume and cortical thickness (Garavan et al., 2002; Moriguchi and Hiraki, 2013; Yasumura et al., 2014; Yuan and Raza, 2014). Furthermore, Davis et al. (2011) found that aerobic exercise intervention contributed to a specific improvement on executive function and activity in prefrontal cortex circuitry in children. Similar findings were also observed in college students. Yanagisawa et al. (2010) suggested that the left dorsolateral prefrontal cortex is likely to be the neural substrate for the improved Stroop performance elicited by an acute bout of moderate exercise. In a recent study, participants exhibited a significant leftward shift of resting prefrontal activation asymmetry after receiving a mind-body exercise treatment (Yasumura et al., 2014). Therefore, besides studying the effect of the BMB intervention on the mood and executive function, we adopted the NIRS measure during the flanker task to investigate the prefrontal activation asymmetry during the congruent and incongruent trial performance induced by an 8-week BMB exercise intervention. Consistent with those previous studies, we found that the BMB training is beneficial for rectifying negative mood and improving executive function, and increasing the left prefrontal cortex activation for the incongruent flanker task, providing some evidence for neural substrate of the improved cognitive performance after a mind-body exercise.

We speculated that mind-body exercise improves executive function via effects on brain systems that underlie cognition and behavior. Executive functions are high-level cognitive functions that subserve and are a prerequisite for self-regulation (Barkley, 2001; Hofmann et al., 2012). Cognitive neurosciences have shown that functioning of self-regulation and executive functions are both strongly but not exclusively dependent on the white matter integrity in the prefrontal cortex (Alvarez and Emory, 2006; Collette et al., 2006). As suggested by Marks et al. (2007), higher levels of aerobic fitness are associated with greater white matter integrity in prefrontal brain areas. The BMB exercise is an easy-to-do aerobic exercise that can improve executive function and brain self-regulation following a period of daily training. Thus, the greater left prefrontal cortex activation during the incongruent flanker task observed in this study may be due to that the mental process of the BMB exercise involves attention and self-regulation (or self-control of cognitive and emotional processes) as core cultivation, and the executive function shares with the brain circuits of self-regulation, mainly in the left prefrontal cortex.

A potential limitation of our study is that participants and exercise coaches cannot be blinded, possibly leading to the performance bias. In addition, the majority of participants recruited in the study were in a narrow age range (between 19 and 26 years), and were relatively well-educated and strong adaptability. Interpretation of the results should be made in light of this. Therefore, it will be very important that future studies are needed to replicate and extend the findings by adopting a larger sample from a wider age range and educational level.

## Author Contributions

CJ and GY designed experiments. TC and YT carried out experiments. TC and GY analyzed experimental results. TC analyzed experimental data and developed analysis tools. TC and CJ wrote the manuscript.

## Conflict of Interest Statement

The authors declare that the research was conducted in the absence of any commercial or financial relationships that could be construed as a potential conflict of interest.

The reviewer RL and the handling Editor declared their shared affiliation, and the handling Editor states that the process nevertheless met the standards of a fair and objective review.
